# Computational and experimental microfluidics: Total analysis system for mixing, sorting, and concentrating particles and cells

**DOI:** 10.1063/5.0158648

**Published:** 2024-04-16

**Authors:** David Coral, Matthew Attard, Eric Pedrol, Rosa Maria Solé, Francesc Díaz, Magdalena Aguiló, Xavier Mateos

**Affiliations:** 1 University Rovira i Virgili (URV), Physics and Crystallography of Materials (FiCMA), Marcel⋅lí Domingo 1, 43007 Tarragona, Spain; 2 SRCiT - Service for Scientific and Technical Resources Campus Sescelades, N2 building, Universitat Rovira i Virgili, Països Catalans 26, Av. 43007 Tarragona, Spain

## Abstract

Body fluids can potentially indicate the presence of non-small cancer cells. Studying these fluids is an emerging field that could be crucial for cancer detection and monitoring treatment effectiveness. Meanwhile, the examination of fluids on a microscopic level is part of the field of microfluidics. This study focuses on the development of a total analysis system that consists of various interconnected structures that are designed to mix, classify, concentrate, and isolate particles in fluids that mimic the behavior of cancer and normal cells. Using the COMSOL Multiphysics software, the device's performance was optimized to use a pressure input of 35 kPa for water or serum and 29.4 kPa for a mixture of liquid and serum samples, which are the optimal pressure inputs. The numerical models were validated by experiments using two types of polystyrene particles, with diameters of 5 and 20 *μ*m. Moreover, the developed system was applied to monitor the behavior of red blood cells. The microfluidic chip is capable of addressing several challenges through visual detections, including mixing tests of two fluids with similar densities, proper particle size classification using Dean flow fractionation, and single-step recovery of large, labeled particles. Finally, the collected particles were examined using an environmental scanning electron microscope to determine their size, and the results demonstrated that successful size separation was achieved, with particles around 20 *μ*m completely separated from the smaller ones.

## I. INTRODUCTION

The accurate diagnosis of diseases is crucial in the medical field, and early detection of prevalent diseases is sought to prevent them from reaching irreversible stages. Genomic analysis has emerged as a promising method for disease detection, but its application is still limited due to ongoing developments in this branch of science. Consequently, patients with detectable discomfort may already be in an irreversible state before the disease can be detected and treated. As a result, extensive efforts have been directed toward developing screening methodologies, which aim to identify diseases in their initial or advanced stages by employing diagnostic methods in a population at risk.^1^ Despite the promise of screening, it is necessary to adhere to specific criteria to ensure accurate testing, as highlighted by Tammemägi *et al.*^2^ and Wilson and Jungner^3^ in their work. They prove the importance of exploring and validating the use of screening tests to facilitate the early detection and treatment of diseases.

Cancer is a pathological condition characterized by genetic alterations in cells caused by intrinsic or extrinsic factors. The World Health Organization (W.H.O.)^4^ has identified cancer as a prominent cause of mortality, with 19.29 × 10^6^ new cases and 9.95 × 10^6^ deaths reported in 2020 alone.^5^ Among the various types of cancers, lung cancer represents 11.6% of all cases worldwide and is responsible for 20% of cancer-related deaths.^6,7^ In 2020, lung cancer accounted for more than 2 × 10^6^ diagnoses, and 1.79 × 10^6^ deaths were attributed to it.^5^ Given the significant burden of lung cancer, it is imperative to develop effective strategies for the early detection of the disease.

Currently, low-dose computed tomography (LDCT) is widely used for detecting lung cancer. The US Preventive Services Task Force (USA) recommends this method for patients aged 55–74 with a smoking history of at least 30 packs of tobacco per year for more than 30 years.^7–9^ The National Lung Screening Trial (NLST) has demonstrated the effectiveness of LDCT in reducing the mortality rate by 6.2% when used as a preventive measure in the initial stages (A1) of lung cancer. Applying this screening test to asymptomatic patients with a high probability of developing cancer results in a significant reduction in mortality.^10^ However, the high false-positive rate is a limitation of this method.^11^ Given that non-small cancer cells have an average size ranging from 16 to 20 *μ*m, while erythrocytes are 6–8 *μ*m and biomarkers are 1 *μ*m, alternative screening tests have emerged. One such approach involves testing blood or saliva; for example, saliva is a human secretion that may contain small concentrations of cancer cells in patients with cancer affectation. Recent studies using Surface-enhanced Raman Spectroscopy have shown positive results in detecting biomarkers associated with breast tumor cells and lung cancer cells in the mixture of saliva and serum.^12–15^ Additionally, researchers such as Wei *et al.* are investigating multiplexable electrochemical sensors to identify mutations in the epidermal growth factor receptor (EGFR) for detecting non-small cell lung carcinomas.^16^

Moreover, body fluids can be studied in a microfluidic system following the branch of the lab-on-a-chip. Within the field of liquid biopsy, by analyzing them in order to detect molecules and particles of interest, the condition of the body could be known. It also contains proteins, DNA, ARN, or microbiotas.^17^ These components might affect the detection of cancer cells by optical methods in microfluidic systems.

The lab-on-a-chip is a microfluidic system that operates within the nano- and micrometer scales, typically below 1 *μ*m or between 10 and 200 *μ*m.^18^ These systems follow the concept of a Total Analysis System (TAS), which aims to integrate multiple chemical or physical analysis steps into a single device.^19–21^ The majority of microfluidic systems can be categorized into three groups: passive systems, active systems, and manual systems. The main distinction between active and passive systems is the introduction of external agents such as temperature, gravity, and electricity, among others, to accelerate or simplify device operation.^22,23^ In contrast, passive devices function independently of automatic pumping systems or electronic equipment, such as the self-sufficient pressure pump developed by Thurgood *et al.*, which utilizes latex balloons.^24^ The detection of particles and cells in a fluid can be greatly facilitated through the use of a microfluidic system. The lab-on-chip technology offers various operational units that can control the physical behavior of particles in a microchannel. The geometry design of the system can enable different unit processes, such as mixing of different fluids,^25,26^ separation of particles within fluids such as cells or plastics,^27,28^ concentration of particles and compounds in a fluid,^28^ and the focusing of particles for subsequent detection.^29^

Therefore, in this work, we present a microfluidic TAS based on visual detection. Our study comprises numerical simulations through COMSOL Multiphysics software^30^ and visualization experiments. This TAS has different coupled geometries that perform mixing and separation throughout a Fermat spiral structure containing grooves to increase the mixing process, the concentration of polystyrene particles, and a focusing part for detection. We also applied the TAS to red blood cells and successfully concluded that this microfluidic device can be used as a preliminary system for screening methods for the detection of non-normal cells, e.g., cancer cells from blood samples.

## II. RESULTS AND DISCUSSION

This section deals with the computational and experimental results achieved in this research. Additionally, for the experimental designs, the thickness of each geometry was measured using confocal laser scanning microscopy (CLSM) where the mixer, separator, concentrator, and full chip yielded the following results 104, 102 , 108, and 98 *μ*m, respectively, which are values that are close to the theoretical, simulated value (100 *μ*m).

### A. Mixer

**FIG. 1.**
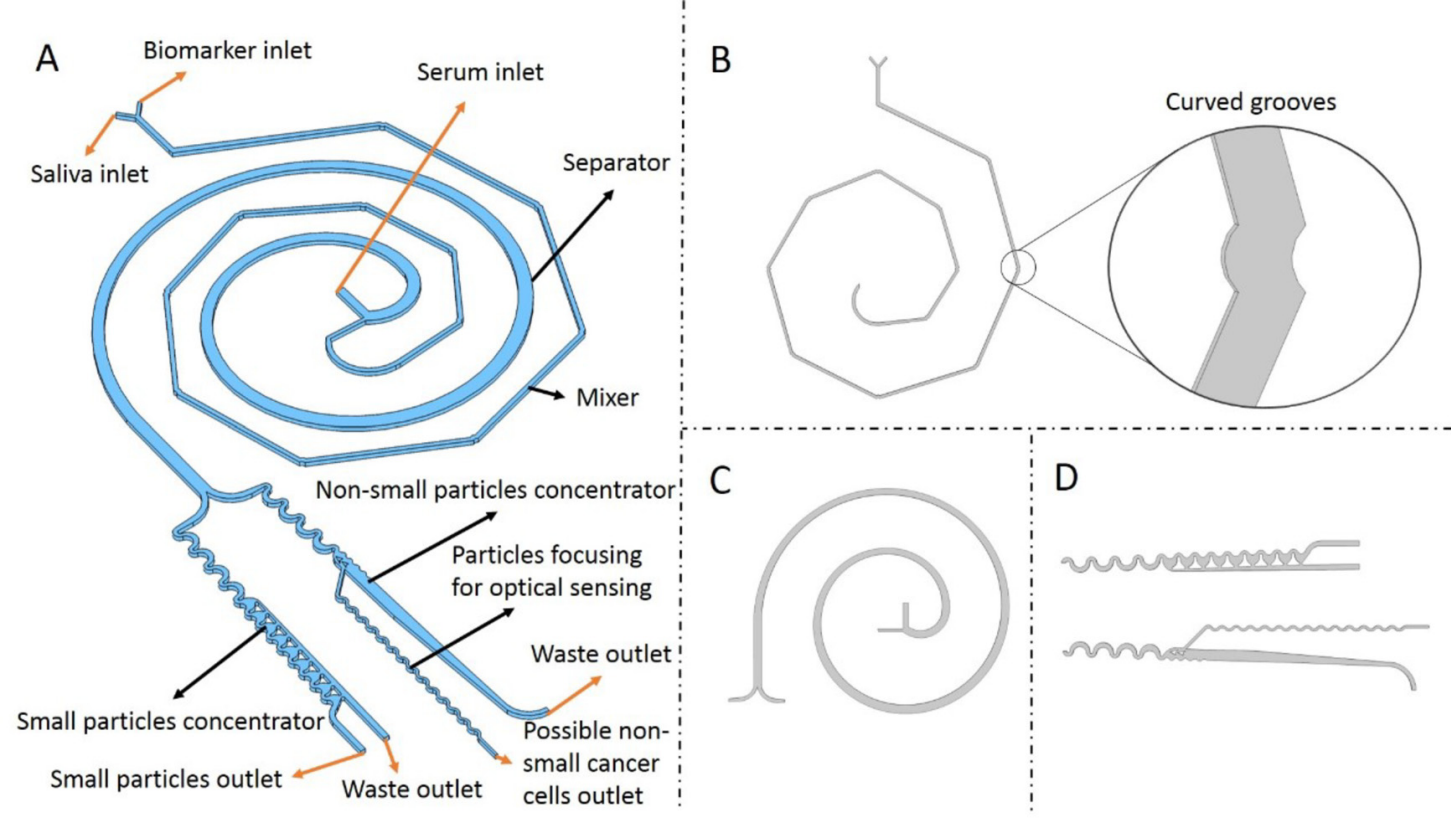
Chip design with coupled geometries: (a) concept design and description of its parts. (b) Mixer, spiral microfluidic geometry, and curved grooves for the mixing of the biomarkers and samples containing cancer cells. (c) Separator, spiral geometry for sorting the biomarkers, small particles, and huge particles. (d) Top: pre-concentrator and concentrator for small particles. Bottom: pre-concentrator for non-small particles.

Among the general chip design [Fig. 1(a)], the first designed geometry is a novel method for achieving efficient mixing, using a system consisting of a Y-junction inlet with grooves that create a spiral by deflecting the channel, forming the first half of the spiral [Fig. 1(b)]. The mixer and separator geometry create a Fermat spiral design. The length and narrow channel are crucial factors for proper mixing. The presence of smooth angles containing the grooves adds additional stress to the fluid, by varying the Dean vortex [as shown in Fig. 1(b)], altering the fluid behavior, and improving the mixing process. The mixing efficiency was analyzed by the homogeneity of the color along the channel. The effectiveness of this approach was previously observed in studies that analyzed serpentine micromixer geometries with different groove shapes.^32^

It is essential to know if this geometry performs adequately over a wide range of pressures, as the rest of the geometries will require more specific parameters to work, and these, in turn, will depend on the pressure and outlet velocity conditions of the mixer. An inherent advantage of incorporating Fermat's spiral into the numerical model is its ability to ensure the successful mixing of two incoming fluids as they enter the separator, irrespective of variables like input velocity and input pressure. Furthermore, beyond what has been mentioned previously, this particular design, when contrasted with alternative spiral or serpentine configurations, exhibits a unique advantage in that it efficiently utilizes the available space within the second part of Fermat's spiral for the integration of additional functional units, like the separator. Moreover, the linear nature of the sections interconnected by the grooves ensures that particles are not subject to separation due to inertial forces, thus allowing diffusion phenomena to play a more prominent role.^26,32,33^

#### 1. Computational model

**FIG. 2.**
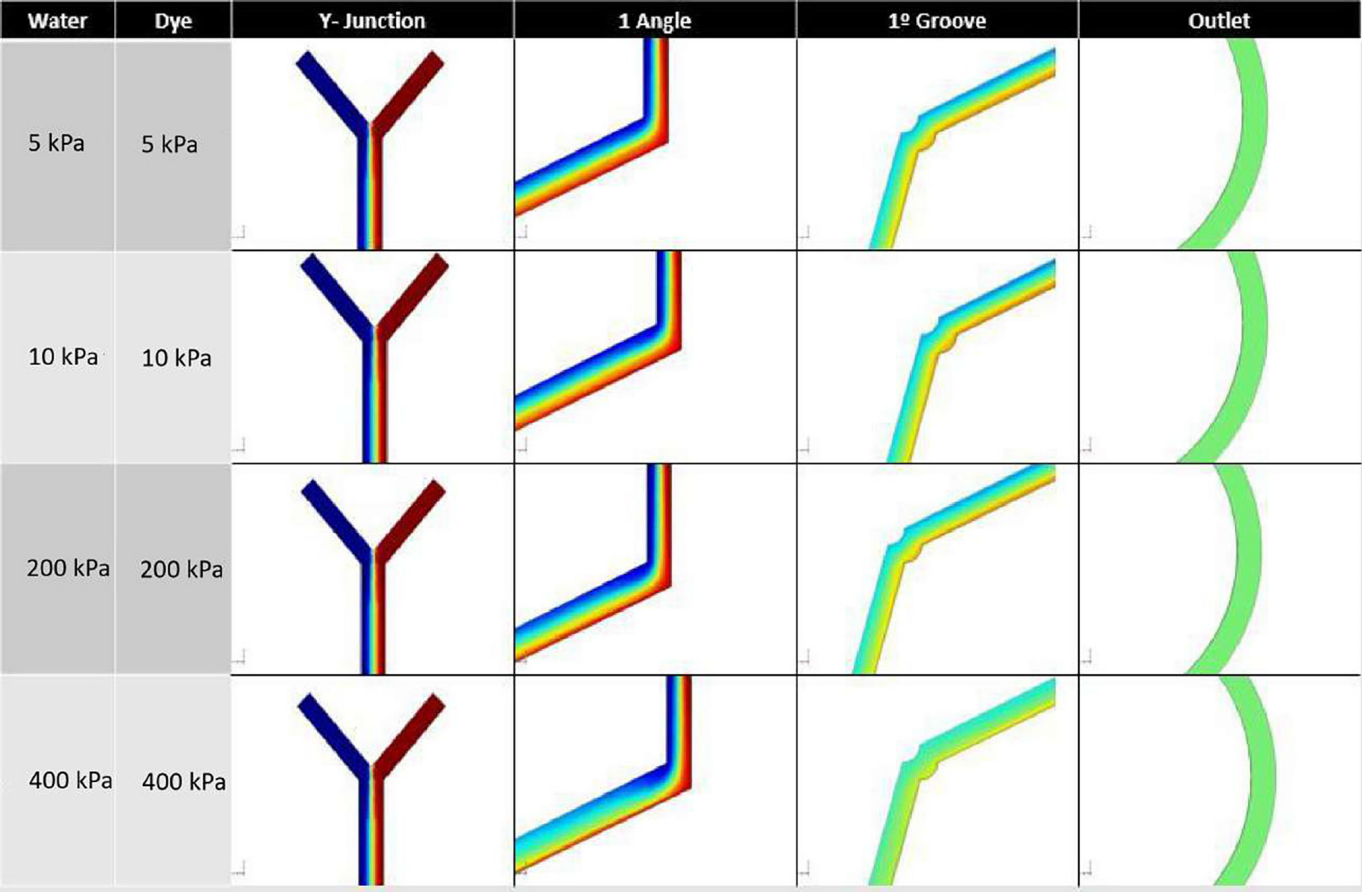
Computational study of the mixer varying the inlet pressure. The left column represents the inlet pressures in kiloPascal. The measurement points are at the Y-junction inlet, the first angle, the first groove, and the mixer output.

Using the COMSOL Multiphysics software, the Navier–Stokes equations were solved for the 3D geometry. A fluid inlet as a function of pressure was conducted, varying the pressure from 5 to 40 kPa as shown in Fig. 2.

As a result, it has been observed that along the geometry from the initial point to the first groove, the fluid is not completely mixed regardless of the inlet pressure. However, there is a mixing trend as the pressure is increased, a phenomenon explained by the turbulence caused at the angulation points. Thus, at pressures of 40 kPa, it is observed that the mixing is more advanced than at 5 kPa. Additionally, at the final point of the mixer, the fluid is completely mixed regardless of the pressure, so it can be concluded that the number of grooves present in the mixer allows the inlet pressure of this geometry to have a wide range of maneuverability. This is a fundamental characteristic when a total analysis system is desired.

#### 2. Experimental model

**FIG. 3.**
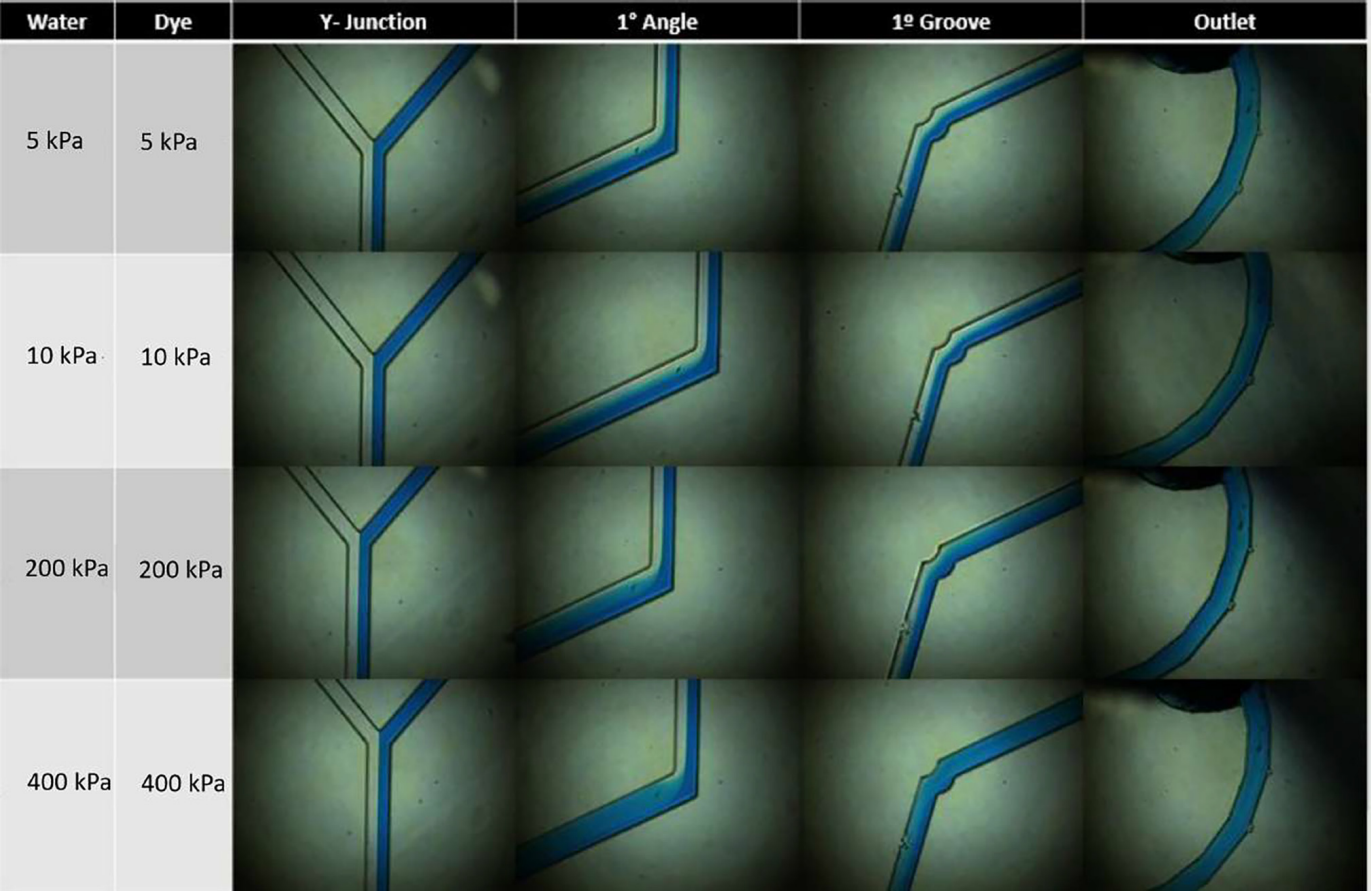
Experimental study of the mixer varying the inlet pressure. The left column represents the inlet pressures in kiloPascal. The measurement points are at the Y-junction inlet, the first angle, the first groove, and the mixer output. As pressure is increased, the fluid mixes earlier within the unit.

To ensure successful separation, it is important for the mixer to have a wide operating range of pressure since the outlet pressure of the fluid in this geometry must be adjusted depending on the conditions of the sample inlet. The Fermat's spiral used in the numerical model has an advantage in that the length, grooves, and narrowness of the channel allow for the successful mixing of the two fluid inlets regardless of the input velocity or pressure. This is corroborated in the pressure study in Fig. 3, where an increase in inlet pressure results in a faster and more homogenous mixing process. The study tested pressures ranging from 10 to 80 kPa, with significant improvements in mixing time observed. These results were validated using a computational model, as shown in Fig. 2.

### B. Separator

The Fermat spiral's second half functions as a separator, with a spiral in the opposite direction to the center of the spiral. This design separates particles by size, considering particle density and size. The sorting process is possible due to the Dean flow, which creates a vortex inside the channel throughout the design.^34^ Different particle sizes were tested in the computational model, and four-particle sizes were selected based on the final purpose of the TAS: 2 *μ*m particles resembling the biomarker attached to a particle to be detected; 6 *μ*m particles simulating non-cancerous epithelial cells; and particles of 15 and 20 *μ*m representing non-small cancer cells. To avoid false positives in visual sensing, unattached biomarkers and possible epithelial cells need to be discarded. Therefore, small particles, such as unattached biomarkers and epithelial cells, are discarded, and large particles are considered cancer cells, as they are the sizes of interest. The sorting geometry's limit size separation is 10 *μ*m. Simulated particles underwent different pressures to vary the velocity and ensure adequate Dean flow, leading to proper size separation. Figure 3 illustrates the curved channel's Dean flow fractionation (DFF) classification of particles.

#### 1. Computational model

**FIG. 4.**
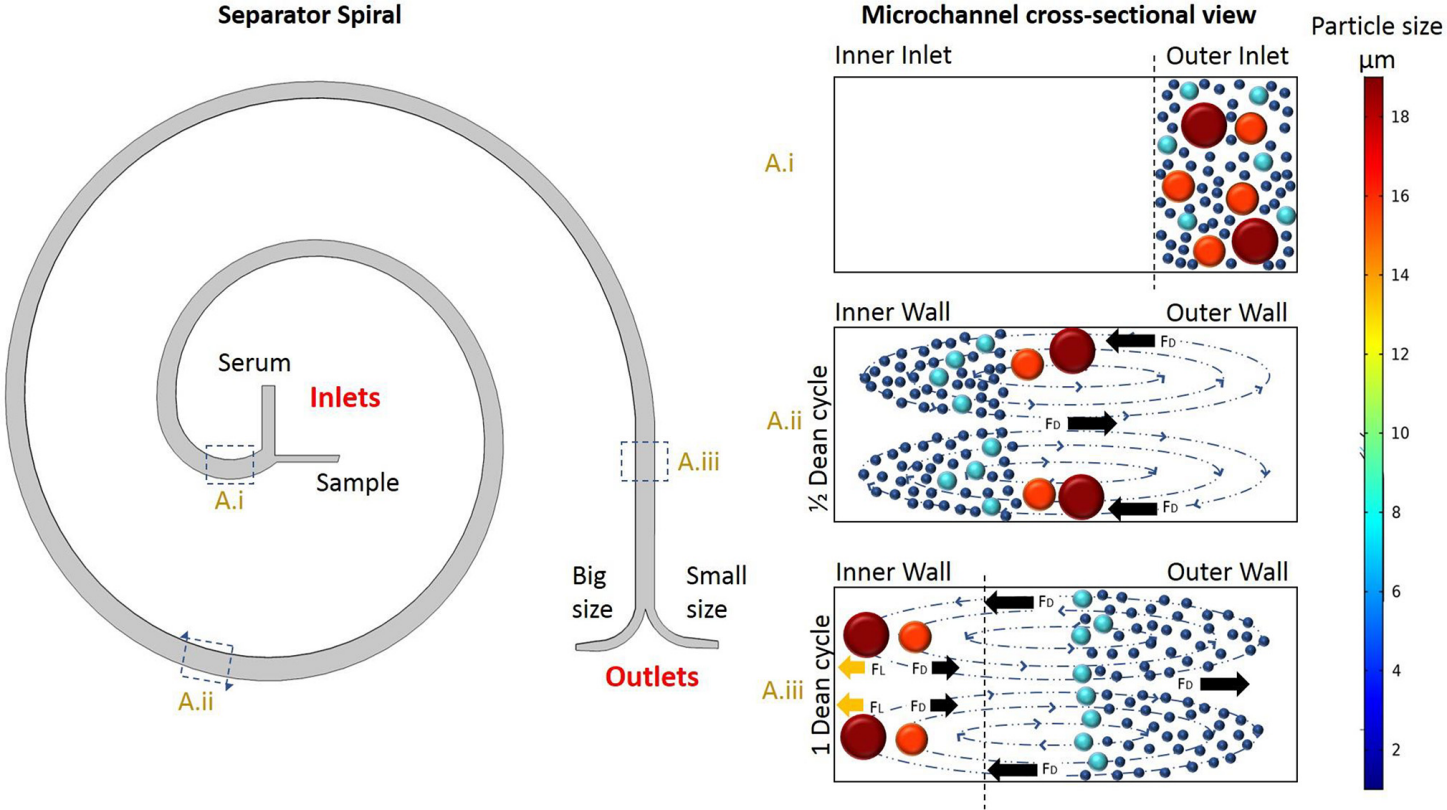
Schematic illustration of the separation principle for high throughput big-sized particles sorting using Dean flow fractionation (DFF). The marked sample and the high-pressure diluting fluid (serum) enter the center of the spiral. (a-i) In the initial part of the separator, the particles enter the system through the outer spiral wall and are confined to the outer wall. (a-ii) Under the influence of Dean drag forces, particles migrate along the Dean vortices to the inner wall. (a-iii) Large particles are confined close to the inner wall where they experience additional strong inertial lift forces (FL) and Dean drag forces (FD). In contrast, smaller particles are influenced to a lesser extent by inertial forces.

**FIG. 5.**
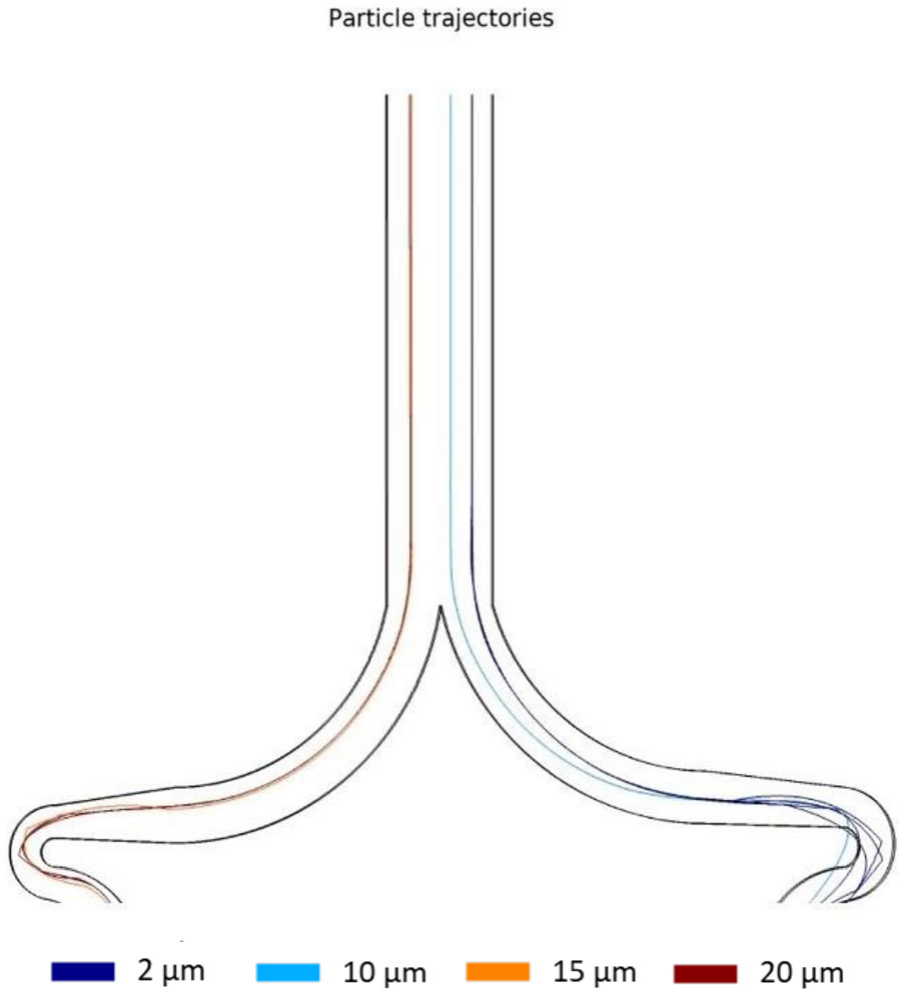
Separator ending. Left stream for the particles above 15 *μ*m and right stream for particles below 15 *μ*m.

The simulation results demonstrated an effective separation process for laminar fluids across a range of conditions. Through multiple simulations, optimal values were identified for achieving an ideal separation. For the fluid containing the sample to be separated (Fig. 4, sample inlet), an inlet pressure higher than 25.2 kPa was found to be optimal, while for the diluent fluid (Fig. 4 serum inlet), a pressure greater than 30 kPa yielded favorable results. It is important to note that achieving confinement at the inlet of the separator requires an inlet pressure ratio of 1.2:1 (serum/sample), as illustrated in Fig. 4(a-i). Deviating from this ratio may result in inadequate confinement at the outer wall of the separator. In Fig. 5, the separation process is depicted with a separator inlet pressure of 35 kPa for the diluent fluid and 29.4 kPa for the sample. Under these conditions, the 15 and 20 *μ*m particles were classified against the inner wall, while smaller particles followed the outer wall. The separation process can be understood in terms of Dean cycles (DC), where particles return to their positions in the cross-sectional plane within the channel after completing each cycle. However, it is important to note that only small particles complete a full DC, as non-small particles reach a point (half a DC) where the dominant forces are lift forces.^35^

#### 2. Experimental model

**FIG. 6.**
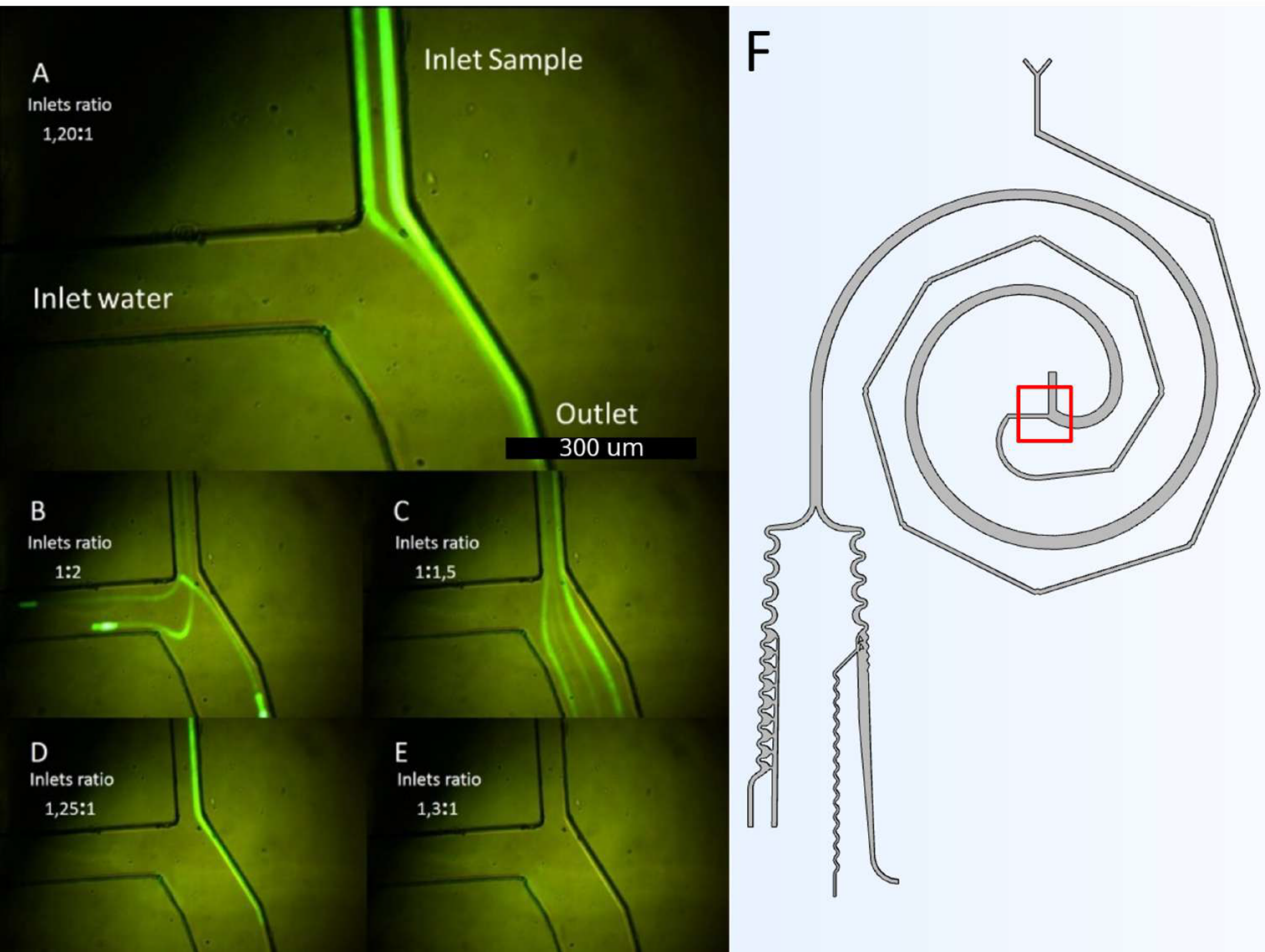
Inlets ratio study to confine the sample. (a) 1.20:1, (b) 1:2, (c) 1:1.5, (d) 1.25:1, and (e) 1.3:1 water to sample experiments. (f) General Scheme and red frame of the represented pictures.

For the experimental procedure, the separator has been tested in two ways: first, by studying the pressure ratio, and second, by studying the pressure for optimal separation. Figure 6 illustrates the results of the pressure ratio study. In Fig. 6(a), the diluted fluid (distilled water) was used with a ratio of 1.2:1 water/sample. The sample stream was confined to the outer wall of the inlet, which is crucial for optimal separation. This behavior is necessary for the best separation process, as explained in Fig. 4. One of the tests studied was the effect of the sample inlet pressure being much higher than the water inlet pressure. Figure 6(b) shows a ratio of 1:2, which directs the sample stream toward the water inlet, preventing it from entering and changing the direction of the water flow. A ratio of 1:1.5 [Fig. 6(c)] causes the water flow to be confined to the inner wall, which does not lead to a good separation process. On the other hand, when the water flow is much higher, water enters the sample inlet, as seen in Fig. 6(e). However, reducing the ratio to 1.2:1 confines the particle flow, and water is backset to the sample stream, as seen in Fig. 6(d).

**FIG. 7.**
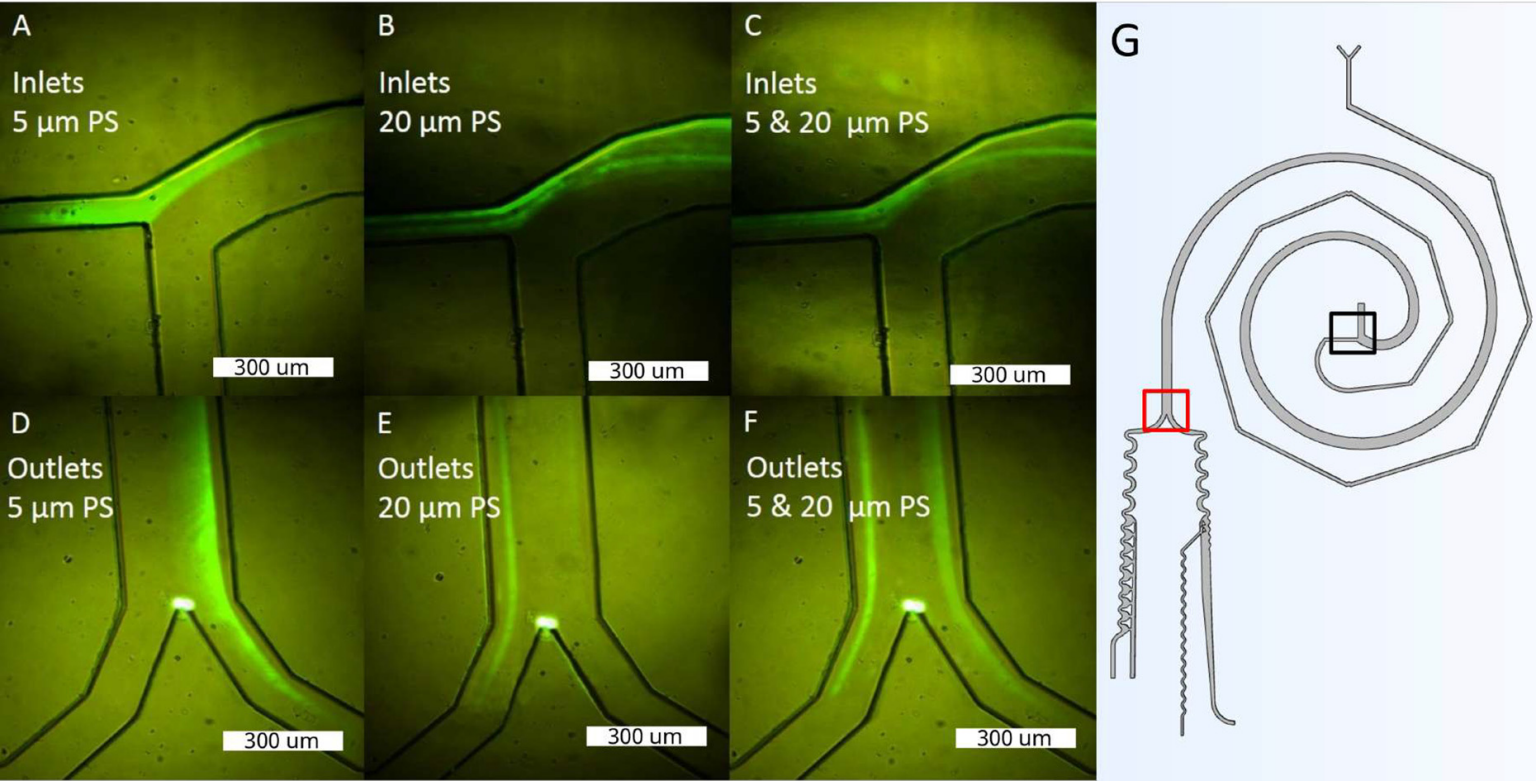
Particles behavior in the separator for inlet pressures of 35 kPa (diluent fluid) and 29.4 kPa (sample). (a) Inlets for the confined particle profile of 5 *μ*m. (b) Inlets for particle profile of 20 *μ*m. (c) Inlets for the mixture of particles between 5 and 20 *μ*m. (d) Outlets with 5 *μ*m particles. (e) Outlets with 20 *μ*m particles. (f) Outlets with a mixture of particles between 5 *μ*m and 20 *μ*m, where the largest particles are correctly separated toward the left exit. (g) General Scheme with a black frame, representing pictures (a)–(c), and a red frame representing the section for pictures (d)–(f).

The study included 14 tests, seven with 5 *μ*m particles and seven with 20 *μ*m particles. The diluent fluid inlet pressure (water) varied from 5 to 35 kPa, and the sample inlet pressure ranged from 4.2 to 29.4 kPa for both cases. The detailed results of the pressure study can be seen in Table S1 of the supplementary material. The optimal pressure ratio of 1.2:1 was maintained for all tests. The same behavior was observed in the numerical model, indicating that optimal separation occurs when the sample inlet pressure is in the ratio of 1.2:1. It was used the most promising pressure values (35 and 29.4 kPa for water/sample), while maintaining the 1.2:1 W/S ratio, to confine the sample stream to the outer wall of the separator inlet and separate the particles at the outlet. Figures 7(a) and 7(d) show the separation of 5 *μ*m particles toward the outer wall of the spiral after completing a Dean cycle under optimal conditions. Similarly, Figs. 7(b) and 7(e) demonstrate the separation of 20 *μ*m particles toward the inner wall of the spiral after completing a Dean cycle. In Figs. 7(c) and 7(f), a sample containing 0.11% (w/v) of 5 *μ*m PS particles, 0.11% (w/v) of 20 *μ*m PS particles, and 99.77% (w/v) water was introduced, resulting in proper sorting of particles by size. The outlet current on the left corresponds to 20 *μ*m particles, while the current on the right corresponds to 5 *μ*m particles, which is consistent with the numerical simulation results shown in Fig. 5.

### C. Concentrator and focusing unit

**FIG. 8.**
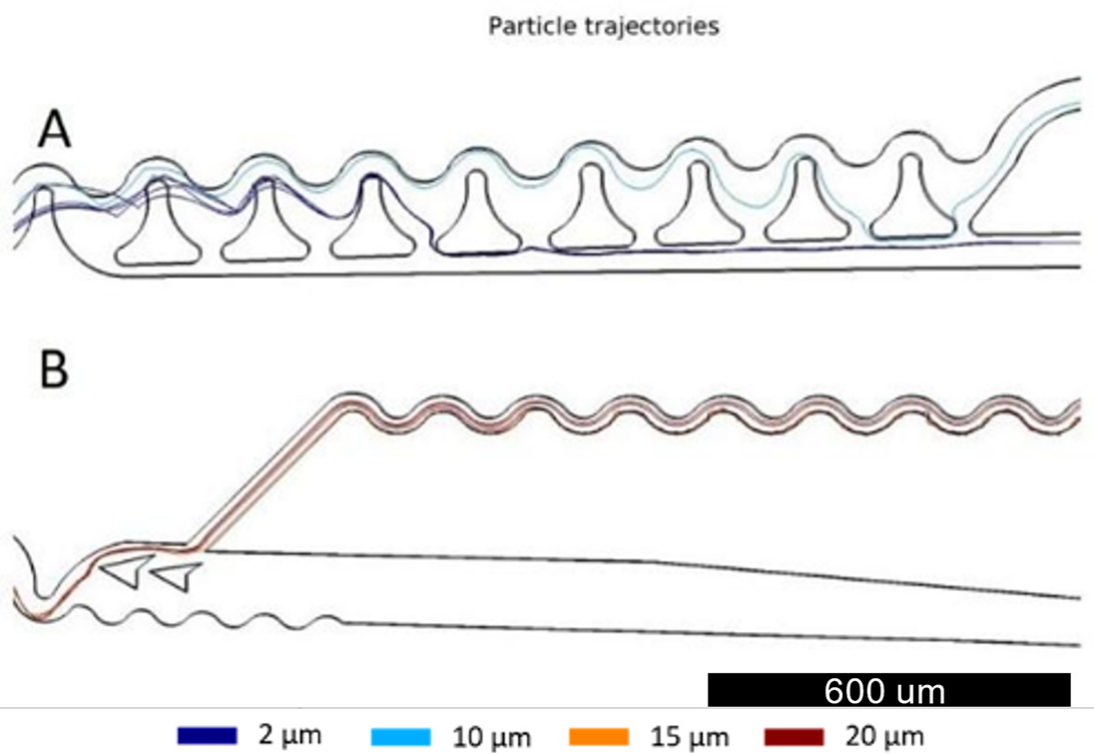
Particle trajectories in concentrator designs. The color scale indicates the particle size. The light blue line represents 2 *μ*m particles, the dark blue line, 6 *μ*m particles, and the red and dark red are 15 and 20 *μ*m, respectively. (a) Small particle concentrator with siphons to remove the liquid for future analysis and to regulate the pressure drop in the system. (b) Large particle concentrator with an expansion to guide particles by momentum and reduce the liquid.

The following computational process was carried out in the concentration unit. For this section, a small particle concentrator Fig. 8 was created to balance the pressure within the system and prevent particles from deviating from their trajectory due to a pressure drop. The continuous flow microparticle concentrator for small particles was designed following the study conducted by Martel and Smith. The focus unit based on inertial volume reduction has a series of siphons to remove the fluid in the particle-free region of the focus units.^36^

#### 1. Computational model

**FIG. 9.**
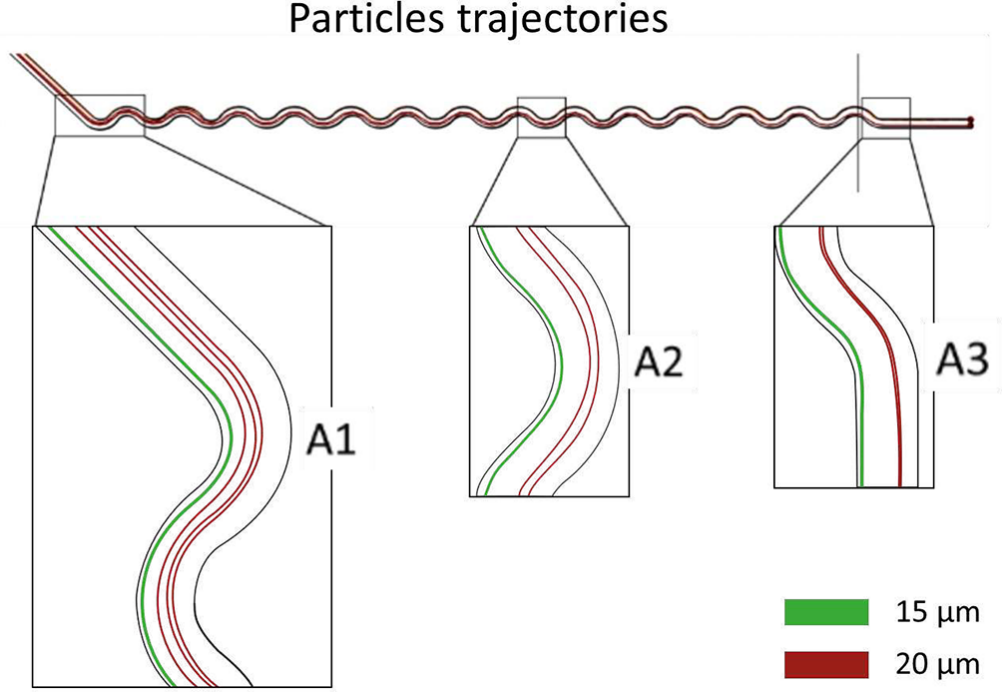
Large particle trajectories for the concentrated stream in the focusing section. (a1) Inlet coupled to the concentrator without aligned particles. (a2) Particles migrating and organizing due to Dean vortex. (a3) Fully aligned particles.

The functionality of the concentrator units is a compromise between the small particle concentrator and the large particle concentrator. As they are coupled to the separator, they depend on the upstream unit exit velocity (the separator). Dimensions other than those established affect the pressure drop of the system as a whole, leading to different particle trajectories. As can be seen in Fig. 5, an optimal selection of conditions for the large particle concentrator [Fig. 8(b)] influences the trajectories in the small particle concentrator [Fig. 8(a)]. This example is a consequence of high flow pressure in contrast to the optimal values required for the small particle concentrator.^36^ One way to solve this problem could be in the length of the final channels in the outlet section, which reduces or increases the pressure drop in that section. However, this could potentially destabilize the entire system. In this work, we focus on the functionality of the large particle concentrator because it is the unit through which possible cancer cells pass. The concentrator functions correctly for pressures above 30 kPa. Inertial forces in the expansion section move larger particles toward the upper wall and expel them through the nearest outlet, causing particles to enter the outlet flow and be confined, as seen in Fig. 8(b) (red flow). Near the upper wall, a bifurcation arises.^35,37^ The narrower section is where the particle focus unit begins, as seen in Fig. 8(b) and detailed in Fig. 9. This unit confines large particles into a single line. This is a result of particle migration along with the Dean vortex and the change of vortex direction caused by the serpentine curvature in the opposite direction.^35,38^ A comparison between the three sections of the serpentine geometry allows further analysis of the confinement effect. In Fig. 9(a1), particles coming from the concentrator decrease their speed due to the high-pressure drop in this geometry. However, particles are not aligned when entering this confiner. Then, in section Fig. 9(a2), particles begin to be influenced by Dean vortices and the increase in fluid velocities; this causes the appearance of inertial forces on the particle, aligning themselves in areas within the channel. Finally, in the last part [Fig. 9(a3)], particles are found aligned in a single beam. It is essential to recognize that this confinement is carried out only in two dimensions; consequently, particles can move freely along the Z-axis (microchannel height).

#### 2. Experimental model

**FIG. 10.**
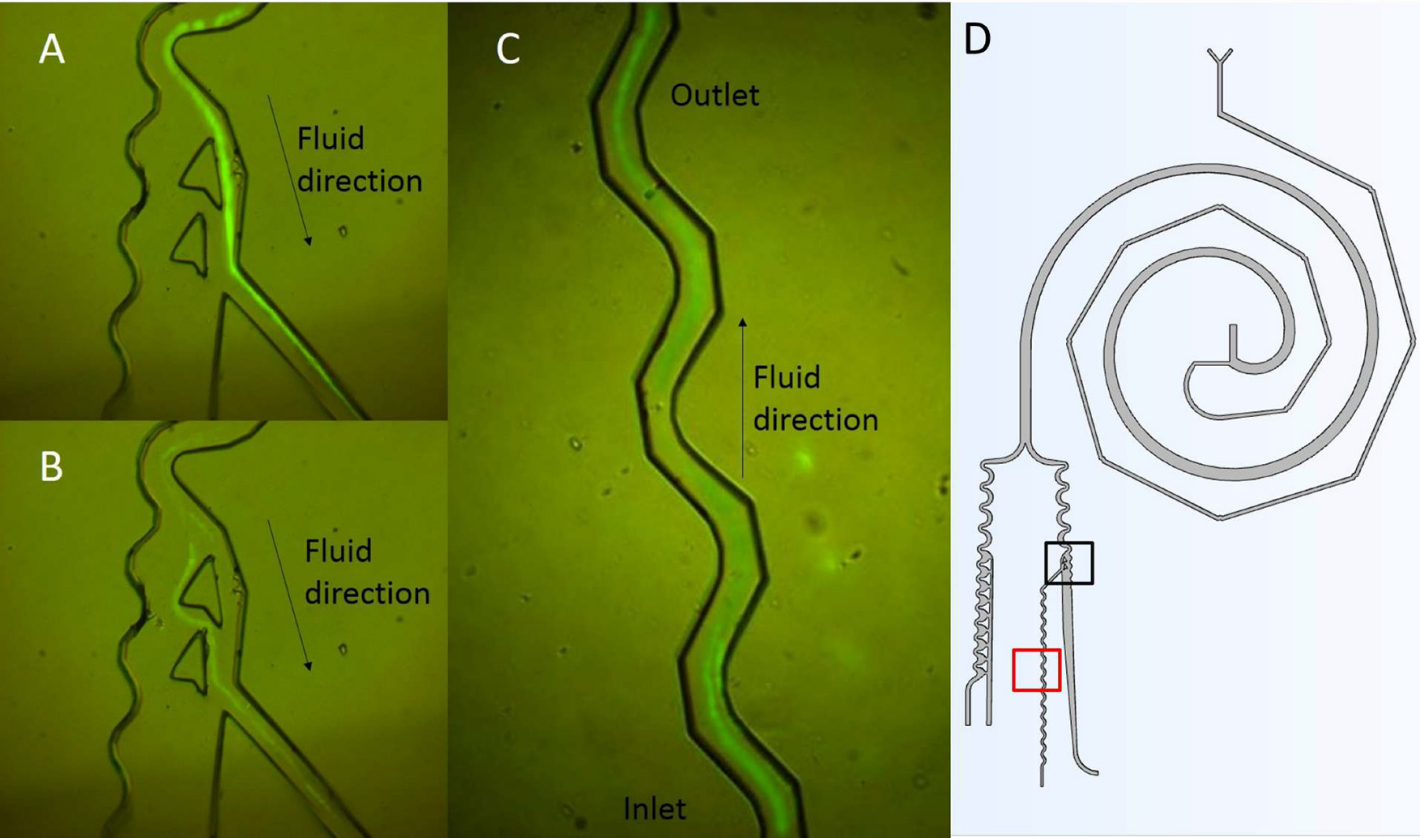
Fluorescent detection of PS particles in the concentrating and focusing unit. (a) Normal behavior with large particles. (b) Deviation in particles trajectory. (c) Focusing unit with PS particles. (d) General Scheme with a black frame, representing pictures (a) and (b). A red frame represents the section for frame (c).

The large particles that makeup the left stream at the end of the separator are composed of particles from 10 to 20 *μ*m, which represent the sizes of non-small cancer cells.^35,39^ In this experiment, we use 0.11% of particles in an aqueous solution. Hence, it is necessary to concentrate the particles to have a faster detection. As can be seen in Fig. 10(a), particles behave in the same way as the simulation in COMSOL Multiphysics [Fig. 8(b)]. However, it is also possible to appreciate additional particle behavior, where they deviate from their trajectory and choose to follow an interruption in the channel [Fig. 10(b)]. In these exceptions, the particle trajectory adopts later the expected path. This new behavior must be related to the pressure of the fluid, as mentioned by authors such as Martel and Smith. The concentrator requires an optimal pressure to make correct confinement of particles.^36^

### D. TAS and ESEM characterization

#### 1. Computational model

**FIG. 11.**
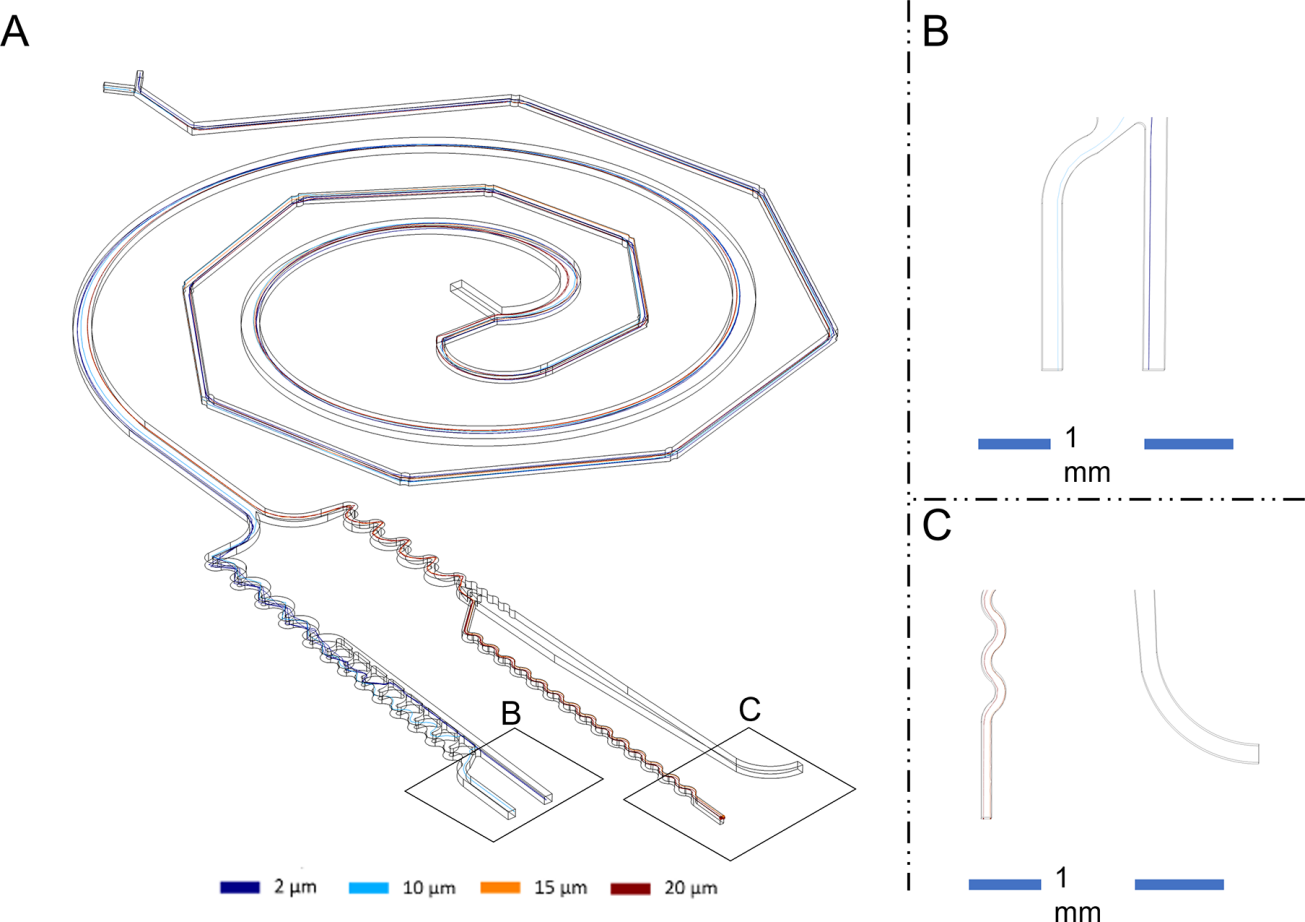
TAS with all geometries coupled. (a) The entire chip illustrates the trajectories of particles, with 2-*μ*m particles marked in dark blue, 10-*μ*m particles in light blue, 15-*μ*m particles in orange, and 20-*μ*m particles in red. (b) Zoom in the outlet stream, for 15- and 20-*μ*m particles. (c) Zoom in the outlet stream for the 2- and 10-*μ*m particles.

Finally, a simulation using the particle tracing for the fluid flow module was done for the full system, as can be seen in Fig. 11(a). Considering all the geometries merged with each other, where particles are mixed, sorted, and concentrated, flinging results like the ones simulated before. The zoom in the outlet indicates the successful operation of the coupled system. As shown in Fig. 11(c), the waste outlet displays no presence of particles, while on the left, particles of 15 and 20 *μ*m (represented by orange and red colors) can be observed. Conversely, Fig. 11(b) shows the particles of no interest, such as 2 and 10 *μ*m. It is important to note that both outlets in Fig. 11(b) serve as waste streams.

#### 2. Experimental model

Once the full system test was conducted, samples were collected from the outlet streams to assess the success of the chip's separation and operation. In order to corroborate the data obtained optically by luminescence analysis, the fluid directed to the “non-small particles” and “small particles outlet” streams [Fig. 1(a)] has been characterized by means of an ESEM analysis. The ESEM images analyzed indicate that specific particle sizes are present in each outlet stream. For the analysis, a 20 kV electron beam energy was employed, and different levels of magnification were applied based on the sample size. It is important to note that, in the case of samples containing small particles, the obtained image exhibited a certain degree of blurriness. This phenomenon can be attributed to the combination of the voltage used and the properties of the sample material.

**FIG. 12.**
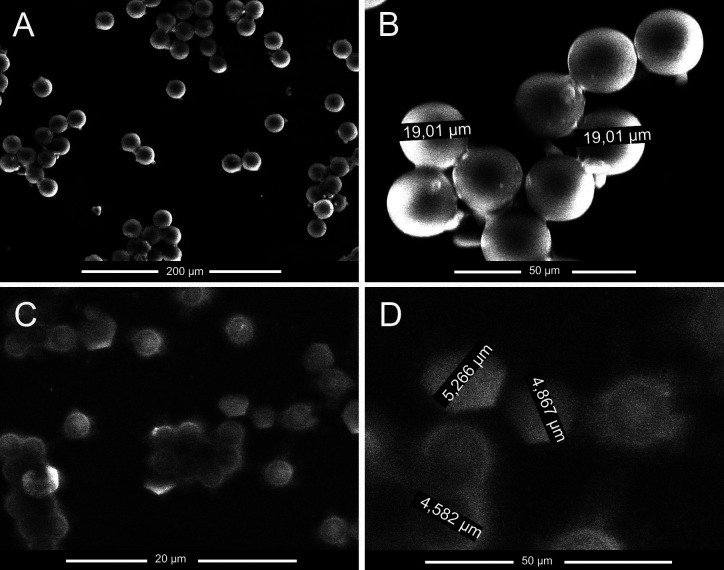
ESEM analysis of the particles collected at the exit of the chip. (a) Dispersion of 20 *μ*m particles in the sample, (b) 20 *μ*m polystyrene particles in the reservoir from the non-small particles stream, (c) dispersion of 5 *μ*m particles in the sample, and (d) polystyrene particles of 5 *μ*m from the small particles stream.

Opting for a higher voltage would degrade the samples, while a lower voltage would reduce the sensitivity of the process. Consequently, the optimal resolution for small samples was found at a magnification of approximately 3900×, coupled with a voltage close to 20 kV. These settings allowed for precise measurement of the polystyrene particles, albeit at the cost of image sharpness. Figures 12(a) and 12(b) depict spherical particles with an average size of approximately 20 *μ*m in the liquid volume collected from the non-small particles outlet. Conversely, Figs. 12(c) and 12(d) show mostly 5 *μ*m particles in the “small particles outlet,” as expected.

These results demonstrate that the particle sizes separated correspond to the simulation data presented, which were experimentally validated by the microfluidic system shown in Fig. 9 during the separation stage through luminescence analysis. The images also confirm that the separated streams are distinct and accurately sorted, and the detected particles by luminescence are the same as the simulated ones.

## III. HUMAN CELLS TEST—RED BLOOD CELLS SEPARATION

One of the potential uses of this Total Analysis System (TAS) is its application in biological studies involving human cells, particularly in the separation of cancerous cells from normal cells, capitalizing on their size differences. This device has the ability to perform this separation passively, thus facilitating subsequent analysis.

**FIG. 13.**
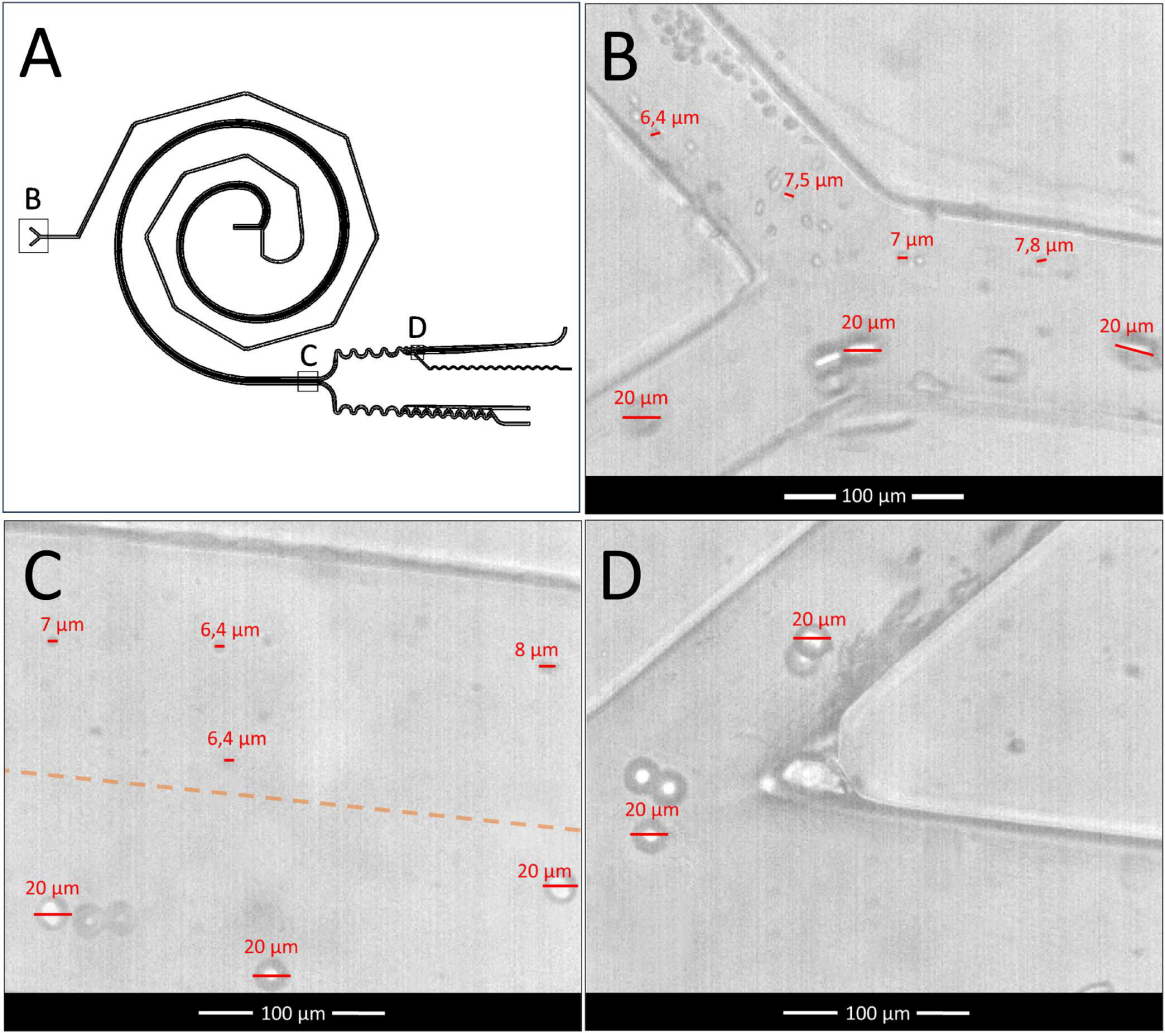
Experimental results from cell testing. (a) The sections of interest. In (b), we observe the entry of red blood cells at the top and 20-*μ*m polystyrene particles at the bottom. (c) The final section of the spiral separator, where the separation of red blood cells and PS particles is achieved at the top. The marked line represents the center of the channel. In (d), the polystyrene particles are directed toward the final bifurcation, ready to enter the stream of interest.

As a result, a series of preliminary tests were conducted to assess the device's behavior when exposed to soft particles, as most previous tests had utilized polystyrene particles. Therefore, red blood cells were employed in these tests, which can deform when subjected to external forces such as variations in velocity and pressure, leading to changes in their shape. In this context, a test was conducted using a mixture of 20-*μ*m polystyrene particles, simulating cancer cells or cells of interest, and red blood cells obtained from the combination of two drops of blood with 3 ml of physiological saline solution. The device received a pre-mixed sample containing particles with an average diameter of 6–7 *μ*m, primarily composed of red blood cells, as depicted in Fig. 13(b). Additionally, a stream of polystyrene particles was introduced while maintaining a pressure ratio of 1.2:1 between the sample inlet and the diluent fluid (35/29.4 kPa), in accordance with the experimental conditions, so each mixer inlet had a pressure of 14.5 kPa.

In Fig. 13(b), a slight deformation of red blood cells can be observed due to the flow velocity. Figure 13(c) shows after passing through the mixer, the particles separate, resulting in two distinct particle streams at the end of this section. In the upper part, there is a stream containing all the red blood cells, while in the lower part, the particles of interest, specifically the polystyrene spheres (down part of the image), are located. Some particles appear different compared to those in Fig. 13(b) because they are positioned below the camera's focal point, giving them a shadowy appearance.

Finally, Fig. 13(d) shows the separation of the 20-*μ*m particles to be introduced into the focusing unit. It can be observed that there is no presence of red blood cells in this area, as they have been separated in the previous stage.

## IV. CONCLUSIONS

Our study presents microfluidic units that could be used in a TAS device for screening body fluids where non-normal cells might exist, such as cancer cells. We defined the necessary functions as a mixer, separator, and concentrator with a focuser unit. We used COMSOL Multiphysics to perform fluid dynamics numerical modeling on each unit to ensure optimal design and functionality for the colloidal system of interest. Additionally, we tested the units experimentally by performing a pressure study on the mixer and separator, built the TAS, and verified the numerical results against experimental observations. Further tests were conducted on the physical separator unit to characterize particle response to initial conditions.

Our proposed mixer unit design combines linear channels linked with grooves following a half-Fermat spiral. Unlike traditional mixer units' designs, our design does not require an increase in channel length when pressure is increased, with the narrowness of the channel and grooves being the primary factors of proper mixing due to their effect on Dean flow. This allows for a wide functionality spectrum.

The separator follows a traditional spiral design and complements the mixer to form a full Fermat spiral, leading to a compact microfluidic functional device. We use a set of a small particle concentrator unit and a non-small particle concentrator-focuser unit. We proposed a novel design for the non-small particle concentrator of continuous fluid flow, which integrates a focuser unit design. Both units are entangled by a trade-off between concentrator functionality and inlet conditions of the set. Future work includes testing with real cancer cells.

Microscopic (ESEM) characterization confirms the proper separation and functioning of the microfluidic chip, demonstrating the successful coupling of various geometries into a single system. The device can mix two streams with particles of different sizes and separate particles of interest (20 *μ*m) from small particles.

To end up with it, as this is a preliminary study involving human cells, it can be concluded that the system is capable of handling cells as if they were polystyrene particles. Therefore, it is expected that in future studies with cancer cells, the behavior will be similar to that of the 20-*μ*m PS particles.

## V. METHODS

### A. Computational modeling

The software COMSOL Multiphysics Ré (COMSOL INC S.A) was used to model all the designs in the different steps, including the final design that merges all the steps [Fig. 1(a)]. In the first step, Fermat's spiral geometry was proposed, where half of the spiral functions as the mixing unit [Fig. 1(b)] with several segments connected using curved grooves, and the other half is the standard spiral geometry [Fig. 1(c)]. To obtain the concentration profiles through the microchannel, sundry simulations were performed using the module Transport for diluting species, and the module Particles Tracing for Fluid Flow was used for sorting particles based on the inertial forces due to size differentiation. The third step involves two units for concentrating particles, as shown in Fig. 1(d). Each set handles different ranges of particle sizes, with the first set receiving particles smaller than 15 *μ*m as input while larger particles are guided into the second set.

The first set is designed following a traditional serpentine concentrator unit. The second set is a modified serpentine unit that handles non-small particles. The modified unit benefits from the momentum carried by non-small particles. The output of this modification produces a concentrated stream, which is then focused using a serpentine unit.

### B. Chip fabrication

The fabrication of PDMS chips followed a standard procedure,^31^ using a silicon wafer (100 mm, Mitsubishi Silicon America) as the substrate. A spin coater (POLOS) was used for layering. To ensure accurate chip measurements, a nanometer-scale mask of the chip has been created using the Direct Write Laser Lithography technique (DWL 66fs—Heidelberg Instruments). The microchannels were fabricated over 3 in. of a silicon wafer, dehydrated for 1 h on a hot plate at 100 °C, and then a spin coating for the structure fabrication was used to prepare the resin for UV exposure. The first layer applied was an adhesion-promoting agent called OMNICOAT (OmniCoat, MicroChem Corp.). Next, the silicon wafer was coated with SU8 resin (SU8 2150, MicroChem Corp). The patterns were transferred from the mask to the SU8 resin through UV exposure using a mercury discharge lamp (MG 1410, Karl Suss). The silicon wafer was developed in PGMEA solvent (AZ® EBR Solvent) to remove excess resin. Finally, a reactive ion etching (RIE) (PlasmaPro NGP80, Oxford Instruments) was used to remove and clean the wafers, leaving only the patterns over the silicon wafer.

The SU8 master was deposited at the bottom of an aluminum container to be coated by PDMS (Sylgard 184, Dow Corning). Previously, a 20 ml mixture of PDMS resin with 10% by weight of catalyst for each SU8 master was degassed for one hour. The mixture was then poured into the aluminum container, completely covering the SU8 master, leaving it to cure for 2 h at 80^∘^C to speed up the polymerization process. The PDMS layer was subsequently removed. Each geometry was established as an independent chip; therefore, the surface of the four PDMS chips was exposed to RIE-generated oxygen plasma to bond the roof layer with the pattern layer under the following conditions: Power: 20 W, oxygen flow rate: 35  sccm, pressure: 50 mTorr, and time: 30 s.

### C. Studied microparticles and characterization

A thorough and detailed evaluation was carried out on each of the geometries, both theoretically and experimentally. In this process, the functionality of each geometry was taken into consideration. To evaluate the mixing, a Triphenylmethane dye dissolved in de-ionized water was used to observe the effect of mixing two substances. For the separator, tests were conducted using particles of different sizes (luminescent polystyrene particles of 20 ± 1 and 5 ± 1 *μ*m in diameter from Microparticles GmbH, producing a mixture of spherical and hexagonal-shaped microparticles) to analyze the effect of the Dean force on their separation. The concentrator was also subjected to similar tests using the same particles, while the focuser consisted of a stage for separating particles of greater interest, followed by a preliminary test using red blood cells. These consist of 0.5 ml of raw blood from one of the coauthor list, who voluntarily provided it and was the only researcher involved in the part of the experiments treating it. This blood volume was mixed with 3 ml of physiological saline solution and carefully shaken.

Finally, a system that integrates all the previously mentioned geometries was evaluated, creating the TAS. This system underwent rigorous experimental tests to determine its effectiveness and efficiency, and then an environmental scanning electron microscope (ESEM) was used to analyze the particles collected at the end of the TAS.

## SUPPLEMENTARY MATERIAL

See the supplementary material for the following files: Figure S1: full chip particle trajectories—this figure illustrates the results of the full chip simulation, with the “particles tracing” module, where the proper functioning of all the individual components was verified once they were connected. Table S1: Pressure study—table of pressure ratio study with the 14 evaluated values in kPa and comments perceived for each test.

## Data Availability

The data that support the findings of this study are available within the article and its supplementary material.
